# Data for understanding the risk perception of COVID-19 from Vietnamese sample

**DOI:** 10.1016/j.dib.2020.105530

**Published:** 2020-04-10

**Authors:** Toan Luu Duc Huynh

**Affiliations:** School of Banking, University of Economics Ho Chi Minh City, 59C Nguyen Dinh Chieu, District 3, Ho Chi Minh City, Vietnam

**Keywords:** COVID-19 pandemic, Risk perception, Socioeconomics, Media attention

## Abstract

This data article describes the risk perception of COVID-190 from 391 Vietnamese respondents aged from 15 to 47 years. These data have been used in Huynh (2020). These data include the socioeconomics, media attention, and risk perception of COVID-19 in Vietnam through a survey conducted on February 1, 2020. In addition, our data might serve as a reference source for further in-depth surveys to understand the risk perception and social media communication across countries.

Specifications table

**Table:** 

Subject	Infectious Diseases
Specific subject area	Econometric models applied to infectious diseases’ epidemiological data to understand the risk perception through socioeconomics and media attention.
Type of data	Table
How data were acquired	Survey
Data format	Data are in raw format and have been analyzed. An Excel file with data has been uploaded.
Parameters for data collection	There is no parameter used for data collection. It is randomized.
Description of data collection	Data were collected from a random sample of an Internet research source, which provided an electronic questionnaire. Data have been collected since February 1, 2020, when the Vietnamese Prime minister officially declared the global and national emergency scenario.
Data source location	Region: Asia Country: Vietnam
Data accessibility	https://data.mendeley.com/datasets/wh9xk5mp9m/3
Related research article	Huynh, T. L. (2020). The COVID-19 risk perception: A survey on socioeconomics and media attention. *Economics Bulletin*, 40(1), 758-764.

## Value of the data

1. Our data are important because this is the first data collection at the primary level to understand the risk perception in the world regarding the COVID-19 pandemic.
2. Researchers, educators, policymakers, and all institutions involved in public health can benefit from our data because by using these data, they can understand the risk perception in Vietnam for the COVID-19 epidemic.
3. These data can be reused for further insights and development of experiments by comparing the cross-country findings or contributing to meta-analysis in the future.
4. These data can be applied in short term and long term because the COVID-19 pandemic is a global emergency.
5. These data are collected carefully from the beginning of the COVID-19 outbreak in February 2020. Thus, it is timely data collection, which is considered as the additional value of our data.

## 1 Data description

Since COVID-19 was declared as a Public Health Emergency of International Concern by World Health Organization (WHO), many research projects are being conducted to understand the different perspectives of this deadly virus. Therefore, this dataset offers an insightful information based on survey data on how Vietnamese citizens perceived the risks of coronavirus spread in the beginning period of outbreaks, particularly on February 1, 2020. In total, 391 Vietnamese people aged 15–47 years were surveyed for COVID-19-related risk perceptions. The platform for data collection is an Internet research tool provided by the University of Economics, Ho Chi Minh City (Vietnam). The total time duration for survey was 20 days, and the survey was conducted in Vietnamese.

Table 1 demonstrates the data characteristics for continuous variables that the questionnaire asked the respondents. The questionnaire used a 10-point Likert scale from 1 to 10 based on Dawes [1]. Table 2 represents the data summary of variables with intervals for answering.

**Table 1:** Data summary of continuous variables.

Variable	Obs.	Mean	Std. Dev.	Min	Max
AGE *(year)*	371	24.48	6.07	15	47
INCOME *(million Vietnamdong)*	313	10.93	10.29	0.4	50
FAMILYMEMBER *(members)*	379	4.42	1.87	0	10
FREQUENCY *(times)*	373	4.98	6.34	0	24
RISKPERCEPTION *(level)*	386	7.65	1.80	1	10
FAKENEWS *(level)*	385	7.89	1.91	1	10
OFFICIALNEWS *(level)*	386	5.87	1.90	1	10

*Notes:* The respondents could skip uncomfortable questions. Therefore, the number of observation varies for each question.

**Table 2:** Data summary of variables with interval answers.

Variables	Intervals			
GENDER	Male (46.80%)	Female (43.73%)	Others (9.47%)	
RELIGION	Non-religion (62.66%)	Buddhism (29.67%)	Christian and Protestant (4.35%)	Others (3.32%)
CAREER	Student (46.80%)	State officer (13.81%)	Private officer (39.39%)	
PROVINCE	North (20.70%)	Central (15.32%)	South (63.98%)	
EDUCATION	High school (10.91%)	University (69.35%)	Post-graduation (19.74%)	
SOCIALMEDIA	Less 1 h (4.65%)	From 1 to 3 h (46.77%)	More than 3 h (48.58%)	
nCOV-SOURCE	Official information (24.55%)	Social media and word-of-mouth (62.40%)	Others (13.04%)	

In total, the questionnaire used in the 2020 survey included 14 questions. On average, it took approximately 10-15 minutes to complete this survey. The data covered COVID-19 risk perception, socioeconomics, and media attention. Our discussion of theoretical backgrounds and development in the further analysis of the survey variables can be found in the associated research article [2] (Table 3).

**Table 3:** Codebook and the related question.

Codebook	Questions
AGE	How old are you?
GENDER	Which gender do you identify with?
RELIGION	Which religion are you belonging to?
INCOME	What is your monthly household income?
CAREER	What is your current career?
FAMILYMEMBER	How many people are you living with in your household?
SOCIALMEDIA	How frequently do you use social media applications? (Facebook/Instagram, etc.)
PROVINCE	In which part have you mostly lived?
EDUCATION	What is your current educational background?
nCoV-SOURCE	In which source are you looking for information about COVID-19?
FREQUENCY	How many times do you actively look for COVID-19 information per an hour?
RISKPERCEPTION	From 1 to 10, to what extent do you concern/worry about COVID-19?
FAKENEWS	From 1 to 10, to what extent do you think that the number of fake news (not confirmed or verified by any official organizations such as WHO, Ministry of Health and so forth) is overwhelming?
OFFICIALNEWS	From 1 to 10, to what extent do you think that the number of official news (confirmed or verified by any official organizations such as WHO, Ministry of Health and so forth) is overwhelming?

## 2 Experimental design, materials, and methods

The questionnaire consists of three parts. The first part includes basic information of respondents. The second part has several questions regarding the use of social media use. The last part includes detailed information about participants’ risk perception of COVID-19 outbreaks in Vietnam. Our research design is mainly based on the theoretical framework [3]. Social and mass media could influence the risk perception [4] because the information might refer to heuristics. Thus, our questionnaire includes social media use behavior, which might contribute to the perceived risk in each sample. The personal characteristics are the potential sources that have an impact on risk perception [5]. These studies are our referred sources to design the questionnaire regarding the COVID-19 risk perception.

As mentioned earlier, the 10-point Likert scale was used to estimate the risk perception from 1 “totally disagree” and 10 “totally agree”. In addition, apart from risk perception, the respondents were asked to consider the level of fake news as well as the overwhelming news of governmental office about COVID-19 in the 10-point Likert scale. In fact, these questions focus on the respondents’ perception of the number of (fake/official) news. Therefore, we asked the participant the level of overwhelming information on the online platform that they used to search for COVID-19 information. Survey data were collected through an anonymous self-administered questionnaire on the Internet platform. We randomly distributed the questionnaires to three regions in Vietnam (North, Central and South). Our data are the primary and first survey conducted about COVID-19 in Vietnam, a developing country. The analysis of our data could be done by STATA and other software to visualize and use for further econometric models. Therefore, follow-up studies might extend our work and enable to determine some helpful features to succeed in the containment of COVID-19.

## Declaration of Competing Interest

The author declares that there are no known competing financial interests or personal relationships that have or could be perceived to have influenced the work reported in this article.

## Appendix Supplementary materials

Image, application 1
