# Supplementary material for: Data for understanding the risk perception of COVID-19 from Vietnamese sample
Source: Data Brief. 2020 Apr 10;30:105530. doi: 10.1016/j.dib.2020.105530 (PMC7171448; doi:10.1016/j.dib.2020.105530)
Supplement: Supplementary file 1 [file mmc1.docx]

**APPENDIX - QUESTIONNAIRE**

**Data for understanding the risk perception of COVID-19 from Vietnamese sample**

The spread of COVID-19 disease causes the severe consequences across the world. So far these is no official research to estimate the perception of Vietnamese citizens on different perspectives: risk, source of information, behaviors in using social media, etc. This research is designed by research group from School of Banking, University of Economics Ho Chi Minh City (Vietnam) to understand the personal behaviors and perceptions of the novel coronavirus. This questionnaire includes 14 questions and takes about 10-15 min to complete. Your collected data will be only used for researching, and not for commercial purpose.

1. How old are you? *(Insert your answer)*

2. Which gender do you identify with?

a. Male b. Female c. Others

3. Which religion are you belonging to?

a. Non-religion b. Buddhism c. Christian d. Others

4. What is monthly household income? *(Insert your answer)*

5. What is your current career?

a. Students b. Government employee c. Private sector employee

6. How many people are you living with in your household? *(Insert your answer)*

7. How frequency do you use the social media applications? (Facebook/Instagram, etc.)

a. Less than 1 hour b. From 1 to 3 hours c. More than 3 hours

8. In which part have you mostly live?

a. Northern b. Central c. Southern d. Other (Overseas)

9. What is your current educational background?

a. High school b. Undergraduate c. Post-graduate

10. In which source are you looking for information about COVID-19?

a. Official information b. Social media and word-of mouth c. Others

11. How many times do you actively look for COVID-19 information per an hour? *(Insert your answer)*

12. From 1 to 10, to what extent do you concern/worry about the COVID-19? *(Scale from 1 to 10)*

13. From 1 to 10, to what extent do you think that the number of fake news (not confirmed or verified by any official organizations such as WHO, Ministry of Health and so forth) is overwhelming? *(Scale from 1 to 10)*

14. From 1 to 10, to what extent do you think that the number of official news (confirmed or verified by any official organizations such as WHO, Ministry of Health and so forth) is overwhelming? *(Scale from 1 to 10)*
